# Destabilisation of T cell-dependent humoral immunity in sepsis

**DOI:** 10.1042/CS20230517

**Published:** 2024-01-10

**Authors:** Kate Davies, James E. McLaren

**Affiliations:** Division of Infection and Immunity, Cardiff University School of Medicine, Cardiff CF14 4XN, U.K.

**Keywords:** Adaptive immune system, Antibodies, B cells, Sepsis, T cells, T follicular helper cells

## Abstract

Sepsis is a heterogeneous condition defined as life-threatening organ dysfunction caused by a dysregulated host response to infection. For some, sepsis presents as a predominantly suppressive disorder, whilst others experience a pro-inflammatory condition which can culminate in a ‘cytokine storm’. Frequently, patients experience signs of concurrent hyper-inflammation and immunosuppression, underpinning the difficulty in directing effective treatment. Although intensive care unit mortality rates have improved in recent years, one-third of discharged patients die within the following year. Half of post-sepsis deaths are due to exacerbation of pre-existing conditions, whilst half are due to complications arising from a deteriorated immune system. It has been suggested that the intense and dysregulated response to infection may induce irreversible metabolic reprogramming in immune cells. As a critical arm of immune protection in vertebrates, alterations to the adaptive immune system can have devastating repercussions. Indeed, a marked depletion of lymphocytes is observed in sepsis, correlating with increased rates of mortality. Such sepsis-induced lymphopenia has profound consequences on how T cells respond to infection but equally on the humoral immune response that is both elicited by B cells and supported by distinct CD4^+^ T follicular helper (T_FH_) cell subsets. The immunosuppressive state is further exacerbated by functional impairments to the remaining lymphocyte population, including the presence of cells expressing dysfunctional or exhausted phenotypes. This review will specifically focus on how sepsis destabilises the adaptive immune system, with a closer examination on how B cells and CD4^+^ T_FH_ cells are affected by sepsis and the corresponding impact on humoral immunity.

## Sepsis

The inflammatory response to infection is a fundamental aspect of immune protection, aiming to rapidly combat the invading pathogen whilst causing minimal damage to the host [1]. Under homeostasis, this is a tightly controlled network, and inflammation wanes following resolution of infection. However, the response is not always proportionate to the threat, and an exaggerated reaction can lead to tissue damage, organ failure, and death [2].

Indeed, sepsis is defined as life-threatening organ dysfunction caused by a dysregulated host response to infection [3]. Sepsis is a heterogeneous condition in which the clinical presentation can vary substantially between patients, in part because it can be triggered by different pathogen types, even though the majority of cases are bacterial [4]. However, in a large proportion of cases, the infectious organism cannot be identified, with many clinical manifestations of sepsis deemed ‘culture-negative’ in routine tests [5–8]. The health and functional state of the immune system plays an important role in dictating susceptibility to sepsis and the subsequent prognosis. Sepsis in vulnerable populations tends to present as a predominantly suppressive disorder due to an already dampened immune system [9]. Patients show reduced capacity to clear the primary infection and indeed any opportunistic pathogens secondary to the initial insult. Such protracted immunosuppression renders patients highly susceptible to nosocomial infections, proving a dominant cause of death. A retrospective trial investigating an association between survival and microbial burden found a significant correlation between late death and positive blood-culture results, particularly regarding opportunistic pathogens [10]. At the other end of the spectrum, some individuals experience a predominantly pro-inflammatory condition which culminates in a ‘cytokine storm’. Commonly regarded as the hallmark of sepsis, such a response triggers a multitude of innate pathways including the complement and coagulation cascades, which in turn release additional pro-inflammatory mediators [11,12]. The resulting endothelial leakage and intravascular coagulation contribute to systemic damage which itself can be life-threatening. This type of response is typical of sepsis in otherwise young and healthy individuals [13]. If the infection is not brought under control, patients frequently experience signs of concurrent hyper-inflammation and immunosuppression [2,14]. This paradoxical phenomenon underpins the difficulty in directing effective immunomodulatory treatment in sepsis.

Sepsis is estimated to be the cause of 1 in 5 deaths worldwide [15], identifying it as a bigger threat to life than cancer. Now recognised as a global health priority by the World Health Organization [16], sepsis can affect anyone with the highest-risk groups including the elderly, the immunocompromised, pregnant women, and also the very young. Indeed, statistics from 2017 have demonstrated that almost half of global sepsis cases occurred in children [15]. In addition, socioeconomic class is one of the greatest risk-factors, with 85% of cases and sepsis-related deaths occurring in low- and middle-income countries [15]. Although intensive care unit (ICU) mortality rates have improved in recent years, 40% of survivors are re-hospitalised within 90 days of discharge, and a striking one-third of discharged patients die within the following year [17]. Half of post-sepsis deaths are due to exacerbation of pre-existing conditions [18], whilst half are explained by a deterioration of health status as a complication of sepsis, recently coined ‘post-sepsis syndrome’. One-sixth of survivors experience post-sepsis syndrome with at least one cognitive, psychological, or physical impairment, and indeed are more prone to recurrent infection, renal failure, and cardiovascular episodes than matched patients hospitalised for other diagnoses [17]. As such, sepsis poses a significant medical and financial burden on healthcare services worldwide, with the National Health Service in the United Kingdom alone estimated to face annual costs of >£1 billion [19]. Although late-mortality and long-term symptoms following sepsis are well-studied, the causes of sequelae are poorly understood [20]. It has been suggested that the intense and dysregulated response to infection may induce irreversible metabolic reprogramming, manifesting in multiple organs. Such alterations may divert metabolism in immune cells, changing how they interact with their microenvironment and respond to subsequent stimuli [21–23].

Prompt intervention is crucial to increase chances of survival. Aside from initial infection control, modulation of the immune system is a key aspect of treatment in sepsis [24]. There have been no major therapeutic breakthroughs in the last 30 years, with current strategies targeting general aspects of the immune system rather than specifically targeting individual elements [25,26]. Although promise has been shown in multiple pre-clinical trials, treatments often fail to advance past the stage of large-scale randomised clinical trials. This failure is due in part to the vast range of disorders with diverse characteristics that are encompassed by the term ‘sepsis’. The resulting inappropriate selection of patients results in treatments that have shown potential in early studies being disregarded. The overall effect poses a huge challenge in translating research to clinical practice. As a dysfunctional response to infection by definition, there is an essential requirement to uncover the mechanisms underpinning the destabilisation of the immune response to infection in sepsis, to explore new targets for drug development and produce effective ways of modulating the immune system long-term post-recovery. Surprisingly, clinical trials blocking excessive inflammation have proved unsuccessful in reducing mortality rates [27]. Instead, recent work has suggested more promise in exploring therapies aiming to restore the activity of ‘exhausted’ or suppressed immune cells [28].

## The adaptive immune system

The immune response to infection by harmful pathogens in vertebrates utilises two main components, the innate and adaptive immune systems, which cooperate to help eliminate the infection and restore homeostasis. The innate immune system provides a rapid defence strategy that responds to infectious insult in a non-specific manner to quickly address the threat [29]. Although a vital first line of defence, the use of pattern- and damage-recognition receptors restricts cells of the innate immune system to recognition of highly conserved microbial structures. Instead, the adaptive immune system supports the initial innate response through the incorporation of cellular (T cells) and humoral (antibodies produced by B cells) components that generate a highly specific response to invading pathogens [29]. In addition, the adaptive immune system is able to establish immunological memory and distinguish foreign antigens from self. Autoimmune conditions with devastating effects may arise through impaired ability to separate self from non-self, demonstrating the power of the adaptive immune system [30,31].

Adaptive immunity is governed by classes of highly specialised T cells and B cells, which develop via a common lymphoid progenitor [32,33]. Both T cells and B cells possess a diverse repertoire of antigen-sensing receptors that are generated through the rearrangement of receptor gene segments during somatic recombination. The process, which occurs in the bone marrow for B cells and the thymus for T cells, gives rise to naïve cells which enter the circulation and peripheral lymphoid tissues to patrol for foreign antigens. Two main types of conventional T cells exist: CD8^+^ T cells which kill infected cells following antigen recognition, and CD4^+^ T cells which support CD8^+^ T cell responses and antibody-generating B cells, amongst other functions [34–36].

In sepsis, a marked depletion of T cells and B cells is observed, correlating with increased rates of mortality [14,37–39]. Such lymphopenia occurs during the onset of sepsis and has been found to persist up to 28 days post-admission to intensive care [40–42]. The majority of sepsis-related deaths occur when lymphopenia is evident, which can persist for years, exposing survivors to opportunistic bacterial infections and reactivating herpesviruses [43,44]. T cells appear to be disproportionately affected by sepsis with CD4^+^ T cells known to decline to levels seen in patients with AIDS [40]. Consequently, B cells tend to constitute a greater percentage of remaining lymphocytes, although this does not necessarily translate to enhanced B cell activity as a combination of sustained inflammation by high antigen-load and cytokine activity results in functional changes to remaining cells [40]. As such, it has been shown that B cells from patients with septic shock lose their proliferative capacity and display a CD21^low^CD95^high^ phenotype associated with B cell exhaustion [45].

**Figure 1:**
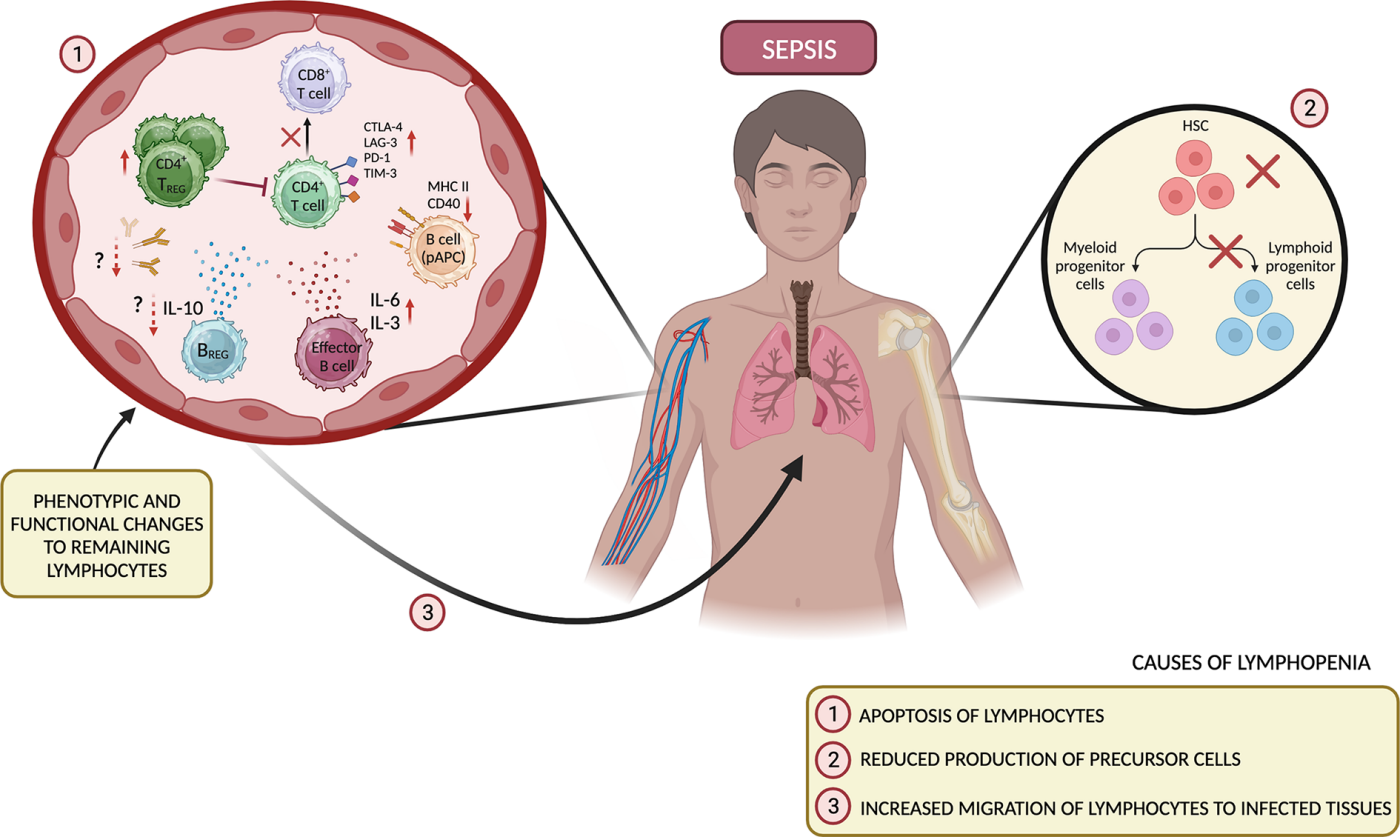
Destabilisation of the adaptive immune system in sepsis A marked lymphopenia is a common feature of patients with sepsis, predominantly attributed to apoptosis of lymphocytes. Other suggested causes include reduced production of precursor cells, and increased migration of lymphocytes to infected tissues, thus reducing the frequency of circulating cells. Remaining cells are reported to exhibit phenotypic and functional alterations, including skewed cytokine production, reduced HLA-DR expression on B cells and increased expression of co-inhibitory receptors on CD4^+^ T cells, which decline in number and provide inadequate help to CD8^+^ T cells. Equally, CD4^+^ T_REG_ cells increase in proportion, but whether this is positively or negatively associated with prognosis has been debated. Furthermore, the benefit of immunosuppression elicited by B_R__EG_ cells is not clearly defined. Immunoglobulin levels decline, but this has been reported to correlate with both improved and worsened outcomes across different studies; HSC, haematopoietic stem cell

The main causes of lymphopenia in sepsis are not fully understood, nor why this can recover in some patients and not in others. Sepsis-associated apoptosis is thought to be a leading cause of T cell and B cell depletion during sepsis [14,37,46–48]. Indeed, post-mortem analyses of spleens from septic patients showed significantly higher levels of caspase-3 activity compared to non-septic patients [46]. Other potential mechanisms underpinning the observed depletion of lymphocytes are relatively understudied but include reduced production of precursor cells. One study reported a significant depletion of haematopoietic stem cells in a mouse model of group A *Streptococcus*-induced sepsis, which was associated with severe immunological stress and early mortality [49]. Additionally, a separate study in humans showed that persistent lymphopenia following cease of initial pro-apoptotic activity correlated with a reduction in common lymphoid progenitor cells caused by osteocyte ablation in septic patients [50]. Alternatively, a reduced pool of peripheral lymphocytes could in part be due to increased recruitment to infected tissues, as has been observed in acute lung injury and chronic inflammatory disorders [51–53]. Such sepsis-induced lymphopenia has profound consequences on how T cells respond to infection but equally on the humoral immune response that is both elicited by B cells and supported by CD4^+^ T follicular helper (T_FH_) cells. The immunosuppressive state is further exacerbated by functional impairments to the remaining lymphocyte population, including the presence of cells expressing dysfunctional or exhausted phenotypes [14,45,54–56] (Figure 1). The majority of studies examining the state of immune dysfunction during sepsis in humans involve analysis of peripheral blood samples, with findings summarised in Table 1. This review will specifically focus on how sepsis destabilises the adaptive immune system, with a closer examination on how B cells and CD4^+^ T_FH_ cells are affected by sepsis and the corresponding impact on humoral immunity.

**Table 1:** Numerical or phenotypic changes to B and T cells in human patients with sepsis or septic shock

Cell	Timepoint	Observations	Reference
**B cells**	**ICU admission**	↓ Combined low serum levels of IgG1, IgM and IgA distinguished patients with highest odds ratio for death	[27]
		↓ Plasma IgG associated with 28-day mortality	[95]
		↓ Frequency of B_REG_ cells associated with increased susceptibility to septic shock and death	[123]
	*+ 28-days post-admission*	↓ Circulating B cells CD40 expression	[41]
		↑ Expression of CD80 and the apoptotic marker CD95 in non-survivors	
	*+ 4- and 8-days post-admission*	↓ HLA-DR expression Circulating B cells	[105]
		↑ Proportional increase in plasmablasts	
		↑ Plasma levels of IgG on day 1, which dropped with time	
	*+ 3- and 7-days post-admission*	↓ Frequency of B_REG_ cells associated with poor outcome, serving as a powerful prognostic marker in elderly patients	[124]
	**Sepsis onset**		
	*+ 2- and 7-days post-onset*	↑ Plasma IgG and IgA on day 1 associated with reduced 90-day survival	
		↑ Proportion of exhausted (CD21^−^/low) B cells	[96]
	*Within 72 h*	↓ Plasma IgM levels, which negatively correlated with severity in elderly patients	[87]
		↓ Capacity for immunoglobulin production when stimulated *ex vivo*	
	*Within 24 h + 24 h post-onset*	↓ Plasma levels of IgA and IgG in non-survivors	[156]
	**Septic shock onset**		
	*+ 3- and 7-days post-onset*	↓ Serum IgM levels, more pronounced in non-survivors	[88]
		↓ Capacity for IgM production when stimulated *ex vivo*	
**T cells**	**ICU admission**	↑ Proportion of Vδ1 T cells, with up-regulation of immunosuppressive co-IRs upon stimulation	[199,200,202]
		↓ Proportion of Vδ2 T cells, with reduced capacity for pro-inflammatory cytokine production Both observations correlated with increased severity and reduced survival	
		↓ Antigen-presenting function of γδ T cells	[203]
		↓ Frequency of MAIT cells	[211,213]
		↑ Markers of activation on remaining MAIT cells along with a reduced cytokine-secreting capacity	
	*+ 4-days post-admission*	↓ γδ T cells, associated with mortality	[200]
	*+ 6-days post-admission*	↓ Percentage of HLA-DR^+^ MAIT cells predicted poor prognosis in patients	[214]
	*+ 5 timepoints up until discharge*	↓ Functional capacity of MAIT cells, which continued to decline with time	[212]
	*+ 3-, 5-, and 7-days post-admission*	↑ Percentage of T_REG_ cells was associated with reduced severity	[181]
	**Sepsis onset**		
	*Within 24 h + 24 h post-onset*	↓ Circulating T_FH_ cells which correlated with increased mortality and low IgA, IgM, and IgG levels	[156]
	**Septic shock onset**	↑ Expression of pro-apoptotic markers, annexin-V binding, active caspase-3 on CD4^+^ and CD8^+^ T cells	[187]
		↑ Expression of PD-1 on CD4^+^ and CD8^+^ T cells, correlated with increased rates of nosocomial infection and death	
	*+ 1-2- and 3–6-days post-onset*	↑ Proportion of T_REG_ cells as a result of a selective depletion of CD25^−^ populations	[177]
	**Post-mortem**	↓ Number and area of lymphoid follicles in patients with sepsis	[37]
		↓ Capacity of splenic and lung T cells to secrete cytokines when stimulated *in vitro*	[14]
		↑ Expression of co-inhibitory receptors	

## B cells

The emergence of adaptive immunity dates back 500 million years, with the added protective value of a specific combinatorial receptor system increasing survivability in vertebrates [57]. Within this time, B cells have evolved several strategies for increasing the diversity of their receptors, enabling identification of almost any antigen [58]. In addition to the initial rearrangement of receptor segments during somatic recombination, B cells increase their receptor variability through processes such as somatic hypermutation, gene conversion, and class-switch recombination [59]. These processes vastly amplify the immunoglobulin repertoire and contribute to a fine-tuned adaptive response. During development in the bone marrow, Pax5 is known to be the master transcription factor behind B cell lineage commitment, acting alongside E2A, EBF1 and IKZF1 [60,61]. Pax5 is a key regulator of many genes important for B cell adhesion and migration (CD55, CD157, CD97, Sdc4, CD44), and signalling (PTEN) [62,63]. This has been demonstrated in Pax5 deficient mice which have a complete absence of mature B cells in the periphery, with a separate study showing ‘dedifferentiation’ of B cells to a common haemopoietic progenitor under conditional Pax5 deletion [64,65]. Immature, ‘transitional’ B cells exit the bone marrow to reach full maturity at peripheral lymphoid sites, completing their development [66].

B cells can be divided into sub-types distinguished by their phenotype and individualised functions [67]. Naïve B cells have traditionally been described either as B-1 B cells, or conventional B-2 B cells, and together they fulfil a range of critical roles in both the innate and adaptive immune system to assist with antimicrobial defence [68]. While the majority of the literature describing B-1 B cells is based on data from mice, a population of CD20^+^ CD27^+^ CD43^+^ CD70^−^ cells has been identified in humans which fulfil key functions characteristic of murine B-1 B cells [69], including the secretion of natural immunoglobulin in the absence of antigenic stimulation [70]. These antibodies have a low affinity for pathogens, but nonetheless confer initial protection in an innate-like response. The role of B-1 B cells in humans remains to be clearly defined. However, they may play an important role in bacterial clearance since a subpopulation of CD5^−^ B-1 B cells can generate antibodies against capsular antigens of *Streptococcus pneumoniae* [71]. To this end, their reported decline with age may play a part in increased susceptibility to infection [69,72].

Conventional B-2 B cells constitute the majority of mature B cells, and are further categorised dependent on their localisation and role [73]. A subset described as marginal zone (MZ) B cells are considered to be innate-like cells, expressing polyreactive B cell receptors (BCRs) capable of binding multiple microbial ‘patterns’ [74]. As such, these cells are strategically positioned in regions prone to frequent microbial exposure such as mucosa and the skin, although circulating MZ B cells have also been reported [75]. Their name describes their predominant localisation to a specialised area of the spleen positioned between the circulation and lymphoid compartment. This region, known as the marginal zone, allows rapid activation of MZ B cells upon interaction with pathogens in the blood [76]. Their importance in bacterial infections is depicted in individuals following splenectomy, with studies reporting increased risk of infection by encapsulated bacteria [77,78]. Their function has been linked to regulation of neutrophil recruitment to the spleen in the early stages of infection, with a study demonstrating MZ B cell-deficient mice to be more susceptible to *Staphylococcus aureus (S. aureus)* infection than wildtype (WT) mice [79].

Although B cells possess the ability to modulate multiple aspects of immune protection through cytokine secretion and their action as antigen presenting cells, they are most commonly associated with their role in antibody production [68]. Follicular (FO) B cells constitute another type of conventional B-2 B cell, occupying the greatest percentage of all B cell lineages. FO B cells differ from MZ B cells through their expression of a highly specific, monoreactive BCR [80]. The fate of precursor cells into FO or MZ B cell subtypes is dictated, in part, by the strength of BCR signalling [81], with stronger signalling favouring precursors to follow the FO B cell differentiation pathway. FO B cells are freely circulating cells that home to secondary lymphoid organs, such as lymph nodes and the spleen, where they may differentiate into plasmablasts or short-lived plasma cells upon activation by antigen [82]. Antibodies secreted by these cells only display moderate affinity for antigen, but nonetheless are important for facilitating early protection [83]. Alternatively, activation may trigger vigorous B cell proliferation, resulting in the formation of specialised microstructures within the B cell follicles known as germinal centres (GCs) [84]. GCs provide the primary site for the interaction of B cells with specialised T cells (i.e. CD4^+^ T_FH_ cells) that support the generation of high-affinity, long-lasting antibodies and memory cells [82]. This system is critical to establish sustained humoral protection against pathogens and underpins the mechanism of protection of most successful vaccines [85]. Under typical conditions, B cells form the foundation of the immune system, modulating the action of other cells through both direct interactions and chemical signals [86]. In sepsis, these relationships come under threat. As the centre of homeostasis, functional changes to B cells offset the entire landscape of the immune system.

## B cells and sepsis

The observed lymphopenia in sepsis appears to be non-homogeneous amongst B cell subsets. Indeed, one study observed a marked plasmacytosis in patients with septic shock compared with healthy controls, which seemingly contradicts the literature reporting decreased concentrations of circulating immunoglobulin [45]. Specifically, the levels of IgM in the sera of sepsis patients have been found to negatively correlate with assessments of disease severity, notably Sequential Organ Failure Assessment (SOFA) and Acute Physiology and Chronic Health Evaluation (APACHE) II scores [87]. Additionally, *ex vivo* stimulated B cells from the same patients displayed reduced capacity to produce IgM [87]. In line with these findings, higher plasma concentrations of IgM within the first 24 h of sepsis have been found to differentiate survivors from non-survivors, highlighting a key protective role of IgM, particularly in fighting Gram-negative infections [39]. Low IgM levels have also been associated with a reduction in the frequency of resting memory B cells, the effect of which was more pronounced in non-survivors [88]. A meta-analysis of studies investigating hypogammaglobulinaemia in sepsis found that as many as 70% of cases experienced low levels of circulating IgG on the day of diagnosis, although an association with clinical outcome remains to be clearly defined [89]. A reduction in general immunoglobulin levels early in infection may, in part, be due to a decline in B-1 B cells. As innate-like producers of natural antibodies, B-1 B cells are suggested to play an important role in compensating for the delay in an FO B cell-mediated adaptive immune response [90]. Early release of low-affinity immunoglobulin by B-1 B cells may infer critical protection in situations where the infectious pathogen has spread to the bloodstream early in infection [91]. The frequency of B-1 B cells has been shown to significantly decline in a murine model of sepsis [92]. The same group found that adoptive transfer of B-1 cells restored IgM levels and significantly reduced lung injury compared with WT mice [93]. In addition to the local and systemic increase in IgM, this result was achieved through attenuation of pro-inflammatory cytokine release and apoptosis, suggesting additional protective roles of B-1 B cells in the response to infection [93]. Sepsis-induced changes to B-1 B cells in humans remain to be characterised but could have therapeutic value if data are consistent with observations in mice.

Despite these findings, the relationship between circulating immunoglobulin levels and mortality in sepsis has proved controversial. Indeed, initial serum IgG levels have been reported to be both positively and negatively associated with clinical outcome [94,95]. A multicentre study measuring IgG_1_, IgM and IgA levels on the first day of severe sepsis or septic shock found that low concentrations of all three antibody types had the highest odds ratio for death [27]. Conversely, the ALBIOS trial found that high IgA and IgG levels at sepsis onset were significantly predictive of both 28- and 90-day mortality [96]. In this trial, low levels of IgG on day 1 were associated with higher risk of secondary infections. These findings again reflect the heterogeneous nature of sepsis, and such variation is likely attributed to subjects experiencing different degrees of inflammation or immunosuppression at the point of testing. Low concentrations of circulating antibodies are indicative of a dampened adaptive response, and so may underpin mortality through a reduced capacity to clear infection. An association between high immunoglobulin levels and mortality in some patients could be explained by the ability of IgG and IgM to activate innate pathways such as the complement cascade, exacerbating an existing state of hyperinflammation through complement-dependent cytotoxicity [97]. Additionally, immune cells such as macrophages, neutrophils and natural killer cells express receptors that bind the Fc portion of antibodies, and so may facilitate the exaggerated host-response through antibody-dependent cellular cytotoxicity and antibody-dependent cellular phagocytosis in the presence of high levels of circulating immunoglobulin [97]. Clearly, gaps remain in defining the association between circulating immunoglobulin and clinical outcome in sepsis. It is likely that there is no clear consensus, and perhaps categorising patients based on a range of clinical observations including plasma immunoglobulin levels amongst other parameters may provide better prognostic value and guidance for treatment.

Beyond antibody production, B cells can also modulate the immune response to infection through their ability to act as a professional antigen presenting cells (APCs) [73]. As professional APCs, B cells are armed with the necessary tools to capture and present processed antigen to T cells. As such, B cells prime and expand antigen-specific T cells, a crucial step for generation of a specific immune response. B cells express both major histocompatibility complex (MHC) I and II molecules, thus enabling them to interact with antigen-specific CD4^+^ and CD8^+^ T cells [73]. In this way, B cells can trigger both T_H_1 and T_H_2 responses to suit the context. One mode of action is through the direct presentation of antigenic peptides to T cells following capture and internalisation of pathogens [98]. Direct presentation is dependent on the antigenic specificity of B cells, defined by their clonotypically expressed BCR. Alternatively, B cells may cross-present free-floating antigen from the extracellular matrix to CD8^+^ T cells [99]. This dual ability is critical for cellular responses against viruses and tumours, where the antigen-presenting B cells are not directly infected.

Following T cell receptor (TCR)-mediated recognition of MHC-restricted antigens on the B cell surface, an immunological synapse is established that promotes T cell activation and drives signals for proliferation, differentiation, and survival. This synaptic connection is strengthened by interactions between co-stimulatory molecules on both cell types, notably CD80/CD86 on B cells with CD28 on T cells [100]. These interactions induce expression of additional costimulatory molecules including CD40 on B cells, as well as adhesion molecules such as LFA-1 and its ligand ICAM-1, that support the process of antigen presentation [101]. Finally, the appropriate effector phenotype is achieved through differential cytokine secretion, polarising the immune response [102]. For example, secretion of interferon-γ (IFN-γ) and interleukin-12 (IL-12) induce signalling cascades which result in T-bet transcription and differentiation towards a T_H_1 phenotype, important for clearance of intracellular pathogens such as viruses and certain bacteria [103]. Secretion of IL-4 induces transcription of GATA-3 and subsequent commitment to a T_H_2 phenotype, important in the response to extracellular infections by parasites and helminths [103]. Other cytokines such as transforming growth factor-β (TGF-β), IL-6, IL-21 and IL-23 support differentiation of alternative helper subsets including T_H_17 cells, and lesser-defined phenotypes including T_H_9 and T_H_22 cells [104]. During sepsis, the expression of MHC II molecules, including human leukocyte antigen-DR (HLA-DR) has been shown to decrease on B cells, altering their ability to present peptides to T cells [105]. This effect has been observed in sepsis patients at the time of admission to ICU and persists in samples taken at a follow-up time of 8 days [105]. A reduction in HLA-DR expression acts to impair the ability for B cells to function as professional APCs, lessening their ability to trigger antigen-specific responses in T cells. In addition, expression of CD40 was significantly reduced on B cells in septic patients at ICU admission compared to healthy donors [41]. No difference in CD40 expression was observed between surviving and non-surviving patients; however, the expression of co-stimulatory molecule CD80 was found to be significantly higher in non-survivors of septic shock at ICU admission [41]. The expression normalised after 3 days, suggesting an enhanced ability to stimulate T cells very early in infection, which perhaps contributes to the hyper-inflammatory state associated with early mortality.

In addition to antigen presentation for stimulation of T cells, B cells themselves can act as cellular effectors [106]. During infection, B cells mediate changes in the inflammatory response through an acquired ability to secrete effector cytokines such as IFN-γ, tumour necrosis factor-α (TNF-α) and IL-17 [107]. Transcriptome analyses in murine models of sepsis show B cells with distinct gene expression profiles, with notable alterations in the expression of genes for several cytokines [108]. In particular, increased expression of pro-inflammatory cytokines such as IL-3, IFN-γ, TNF-α and IL-6, and reduced expression of anti-inflammatory cytokines such as IL-10 and TGF-β1 [108]. In addition to driving systemic inflammation, secretion of cytokines can polarise T cells towards specific helper phenotypes as detailed above [103]. In a murine caecal ligation and puncture (CLP) model of sepsis, B cell deficient (µMT) mice showed reduced concentrations of inflammatory cytokines in sera compared with WT mice, which was not replicated in T cell deficient (TCR αβ^−/−^) mice [109]. These data indicate a role of B cells in triggering an early inflammatory response in sepsis, with further experiments showing the importance of such cytokine production on successful bacterial clearance. Splenic MZ B cells have been shown to produce large quantities of IL-6 and the chemokine CXCL10 after lipopolysaccharide (LPS) challenge *in vivo* in mice [110]. The significance of such a pro-inflammatory response was investigated in mice lacking IL-6-producing MZ B cells (MZ B-IL-6-KO). These mice produced significantly lower amounts of serum IL-6 and CXCL10 and demonstrated improved survival compared with WT mice [110]. Furthermore, administration of an anti-IL-6 receptor (IL-6R) antibody shortly following intravenous injection of *Escherichia coli* (*E. coli*) or the induction of CLP resulted in prolonged survival compared with mice treated with a control antibody [110]. These results indicate a pathogenic role of IL-6 in exacerbating endotoxic shock in sepsis. This finding does not contradict earlier findings that IL-6 plays an anti-inflammatory role very early in sepsis [109], as injection of anti-IL-6R at time-points concurrent with LPS or *E. coli* injection did not affect the survival of mice. At the very early stages of sepsis, IL-6 production by B cells may not augment the inflammatory response to toxin, with delayed onset of its pathogenic role. In addition to IL-6, IL-3 production by B cells in a mouse model of abdominal sepsis has been reported to potentiate inflammation through enhanced production of monocytes and neutrophils, with IL-3 deficiency inferring protection [111]. These findings correlated with observations in humans showing an association between high plasma IL-3 levels and mortality [111]. Despite the reported pro-inflammatory signatures of B cells in sepsis, strategies aiming to modulate cytokine levels have failed to prove beneficial [112]. Patterns of cytokine release change throughout the course of disease, and so timing of administration is likely an important consideration for these types of therapies [109]. Investigations into IL-6 blocking early in infection still show promise [113].

## Regulatory B (B_REG_) cells

B_REG_ cells represent a specialised subtype of B cells that can suppress T cells and the action of other pro-inflammatory cells through the production of IL-10, IL-35 and TGF-β [114]. B_REG_ cells, constituting less than 1% of PBMCs in humans, show heterogeneity in the expression of surface proteins and indeed may differentiate into distinct subsets dependent on the inflammatory stimuli to which they are exposed [115]. For example, studies have reported CD19^+^CD25^hi^ B_REG_ cells that support T regulatory (T_REG_) cell function *in vitro* in co-culture experiments, but also several populations of B_REG_ cells which suppress an anti-tumour response in cancer such as those expressing granzyme B in solid tumour infiltrates, and CD19^+^CD24^+^CD38^+^ cells in breast cancer [116–118]. It is generally accepted that their suppressive ability is enhanced under highly inflammatory conditions to limit further damage, for example, in the case of autoimmune conditions [119–121]. Although sepsis is generally characterised by a protracted lymphopenia, the balance of subsets within the total population of B cells is disturbed. In a CLP model of sepsis in mice, an increase in the frequency of B_REG_ cells was one of the first observable changes, exacerbating an immunosuppressive state [122]. Conversely, B_REG_ cells can play a protective role, with reduced number and function correlating with the development of severe septic shock in mice exposed to endotoxin [108]. Human patients with sepsis have decreased numbers of B_REG_ cells compared with controls, with frequency negatively correlating with likelihood of septic shock [123]. In fact, the levels of B_REG_ cells over the first week post-admission to ICU appear to have particular prognostic value in elderly patients with sepsis [124]. The same was observed in neonates, with an increase in B_REG_ cells positively correlating with survival [125]. Following the onset of septic shock, there is an increase in cells expressing a B_REG_-like cell phenotype, and an associated increase in IL-10 production mirroring the observed immunosuppressive state [45]. Together, these findings suggest a protective role of the immunosuppression elicited by B_REG_ cells early in sepsis, perhaps aiding against deaths caused by overwhelming inflammation and consequent septic shock. In surviving patients, however, B_REG_ cells may tip towards a pathogenic function through continued promotion of an immunosuppressive state in the midst of other cells becoming anergic and unable to respond to subsequent stimuli.

## The potential of B cells in clinical practice

Given the numerical and functional changes exhibited by B cells during sepsis, and the association of certain alterations with morbidity and mortality, it is unsurprising that B cells have been the focus of several studies investigating prognostic biomarkers and therapeutic targets. For example, one group suggested that a low percentage of CD23^+^ B cells at ICU admission enables discrimination between survivors and non-survivors with a sensitivity of 90.9% [41], whilst another demonstrated poor prognostic survival outcome in patients with low IgM levels within the initial 24 h of sepsis onset [126]. In terms of treatment, supplementation of specific B cell subsets that are depleted or dysfunctional during sepsis may restore immune function. For example, adoptively transferring B-1 cells could replenish natural immunoglobulin and suppress excessive inflammation [92,93]. Although levels of circulating immunoglobulin have proved controversial in dictating disease course, considerable attention has been given to the use of intravenous immunoglobulin (IVIG) as an approach to modulate inflammation in sepsis, particularly in neonatal cases [127]. Although IVIG therapy is an approved treatment for multiple conditions of immune dysregulation, including Kawasaki disease which is often difficult to differentiate from sepsis during the early stage of onset [128], IVIG has proved unsuccessful in reducing mortality in several large randomised controlled trials of patients with sepsis [129–132]. Potential limitations to trials include choice of subjects and timing of treatment; with discrepancy in the literature reporting circulating immunoglobulin levels and prognosis in patients with sepsis, treatment needs to be more specific and tailored to the individual. A method of first identifying the state of immunosuppression in patients may enable guided selection for trials, and generate more promising results [133]. The failure of clinical trials has resulted in guidance against the use of IVIG in sepsis and septic shock. Despite this, several studies have reported benefits of IgM- and IgA-enriched immunoglobulin administration [134] and indeed, such preparations are widely used in addition to other treatments in septic shock to enhance immune function [135]. The potential benefit of their combined administration has been suggested to stem from their dual action in both the bloodstream and mucosal surfaces. The overarching consensus for best clinical practice remains a personalised approach, with guidelines for dosage and timing of administration highly dependent on the clinical phenotype.

## CD4^+^ T_FH_ cells

The process of pathogen-specific antibody production is reliant on help signals provided by specialised CD4^+^ T_FH_ cells, which interact with B cells in the GCs of secondary lymphoid organs [136]. GCs provide the primary site for high affinity antibody production via somatic hypermutation and class switching of B cells [84]. CD4^+^ T_FH_ cells govern the movement of B cells throughout the GC, and determine which cells are selected for differentiation into long-lived plasma cells and memory B cells. Not only are CD4^+^ T_FH_ cells crucial for supporting B cells, they play a critical role in GC formation and maintenance [84]. CD4^+^ T_FH_ cells were first described in the early 2000s, following work observing a unique CXCR5^+^ subset of CD4^+^ T cells in tonsillar tissue [137,138]. These cells were shown to express several markers important for B cell activation, indicating their involvement in tonsillar immune responses. Co-culture with naïve B cells demonstrated their capacity to induce class-switched antibody production, which was replicated and built-upon in subsequent studies [139]. However, at this time, CD4^+^ T_FH_ cells were not widely accepted as being distinct from T_H_1 or T_H_2 cells as the transcription factor driving their differentiation was unknown. Years later, CD4^+^ T_REG_ and CD4^+^ T_H_17 cell types were characterised, based on the identification of lineage-determining transcription factors for these populations (FOXP3 for T_REG_ cells and RORγt for T_H_17 cells). It was not until 2009, when the discovery of BCL-6 as a transcription factor essential for GC generation and high affinity antibody production allowed recognition of these cells as an individual CD4^+^ T cell type, acknowledging their distinct role as follicular B cell helpers [140–142].

The GC is divided into two compartments described as the light zone and dark zone, so called due to their histological appearance [84]. These zones form distinct sites for separation of the steps involved in the GC reaction. Within the light zone, B cells present antigen-MHC class II complexes to CD4^+^ T_FH_ cells. In return, select B cells receive co-stimulation and survival signals from CD4^+^ T_FH_ cells to encourage migration to the dark zone. Such signals include IL-21, IL-4, and IL-10 secreted by CD4^+^ T_FH_ cells [143,144]. IL-21 induces transcription of activation-induced cytidine deaminase in B cells, an essential factor for somatic hypermutation [145]. This process involves the introduction of BCR point mutations to generate cells with a range of affinities for antigen. The somatically hypermutated B cells then return to the light zone, where those with highest affinity for antigen are positively selected for proliferation and survival. Further signalling via co-stimulatory molecules, IL-21, and IL-4, initiates their return to the dark zone for isotype class-switching [84]. Class-switched B cells may then either differentiate into plasma cells to secrete high-affinity antigen-specific antibodies or instead become long-lived memory B cells. After fulfilling their role, CD4^+^ T_FH_ cells leave the GC and may either enter a GC in a neighbouring follicle, or re-enter the same GC. Alternatively, CD4^+^ T_FH_ cells may downregulate BCL-6 and enter the blood stream as memory CD4^+^ T_FH_ cells.

Expression of inducible co-stimulator (ICOS) on CD4^+^ T_FH_ cells is important for all stages of differentiation and maintenance. Initially, ICOS on pre-CD4^+^ T_FH_ cells binds to ICOS ligand (ICOSL) on dendritic cells to initiate priming and migration towards the B cell zone of the GC. Later, ICOS/ICOSL signalling between CD4^+^ GC-T_FH_ cells and B cells ensures maintenance of CD4^+^ T_FH_ cells for supporting antibody production. Other markers essential for CD4^+^ T_FH_ cell function include OX40 and CD40 ligand (CD40L). Expression of both proteins is up-regulated following activation of CD4^+^ T_FH_ cells, promoting their accumulation at the T-B border where they bind their ligands on cognate B cells [146,147]. Bidirectional signalling results in IL-21 secretion to assist with B cell activation and proliferation, and GC maintenance [148].

Tight regulation of the GC reaction is necessary to prevent generation of autoantibodies [149,150]. A fine balance is required to enable effective humoral immunity, whilst maintaining self-tolerance. One arm of control is achieved by a specialised subset of CD4^+^ T_REG_ cells known as T follicular regulatory (T_FR_) cells [151]. CD4^+^ T_FR_ cells are similar to CD4^+^ T_FH_ cells in that they express BCL-6 and CXCR5 but are distinguished by their expression of FOXP3. CD4^+^ T_FR_ cells suppress both CD4^+^ T_FH_ and B cells to regulate the GC reaction [128,152]. The mechanisms underpinning suppression remain to be completely elucidated, but one known method involves expression of the co-inhibitory receptor cytotoxic T lymphocyte-associated antigen 4 (CTLA-4), which functions to dampen co-stimulatory interactions between cognate CD4^+^ T_FH_ cells and B cells [153]. In addition, CD4^+^ T_FR_ cells suppress IL-21 and IL-4 transcripts in CD4^+^ T_FH_ cells, two cytokines vital for the selection of high-affinity antibodies in the GC [154].

## CD4^+^ T_FH_ cells and sepsis

Although multiple studies have reported defects in humoral immunity in cases of severe infection and sepsis, these have largely focussed on B cells and alterations in immunoglobulin release [37,41,155]. For patients showing reduced levels of circulating immunoglobulin, proposed mechanisms include an impaired activation-capacity of plasmacytes, with increased expression of markers indicative of an exhausted phenotype [82]. Secondary lymphoid organs from septic patients have been demonstrated to have a lower cellular density than those from healthy controls, encompassing the total follicular B cell population, but also follicular dendritic cells and CD4^+^ T_FH_ cells [37,156]. These findings are consistent with a decline in circulating CD4^+^ T_FH_ cells, and correlate with reduced B cell numbers and increased mortality [156]. Despite these findings, a mechanism whereby impaired B cell maturation could be attributed to changes in the CD4^+^ T_FH_ cell population has yet to be determined. Considering the close relationship between B cells and CD4^+^ T_FH_ cells in the GC, and the dependency of follicular B cells on signals from CD4^+^ T_FH_ cells for proliferation and survival, it seems plausible that a lacking humoral response could stem from insufficient support. Data from a murine model of sepsis showed blunted differentiation and class-switching of B cells in septic mice compared to controls, with reduced expansion and differentiation of CD4^+^ T_FH_ cells following immunisation [157]. Additionally, the importance of CD4^+^ T_FH_ cells in supporting an antigen-specific B cell response has been demonstrated in ‘immune educated’ mice which, compared to standard laboratory mice, present a diverse repertoire of memory T cells [158]. Following induction of CLP-induced sepsis, increased IL-21 production was indicative of increased functionality in CD4^+^ T_FH_ cells, which in turn were able to reverse the sepsis-induced decline in splenic B cells seen in controls. Such an effect was accompanied by enhanced follicular B cell and GC development [158]. These results demonstrate the critical role of CD4^+^ T_FH_ cells in supporting antigen-specific B cell responses in conditions of inflammation. The commonly observed alterations in B cell development and functionality reported in humans suggest a potential defect in this relationship in sepsis. A lack of functional CD4^+^ T_FH_ cells could induce apoptosis of B cells, through a loss of BCR signalling.

**Figure 2:**
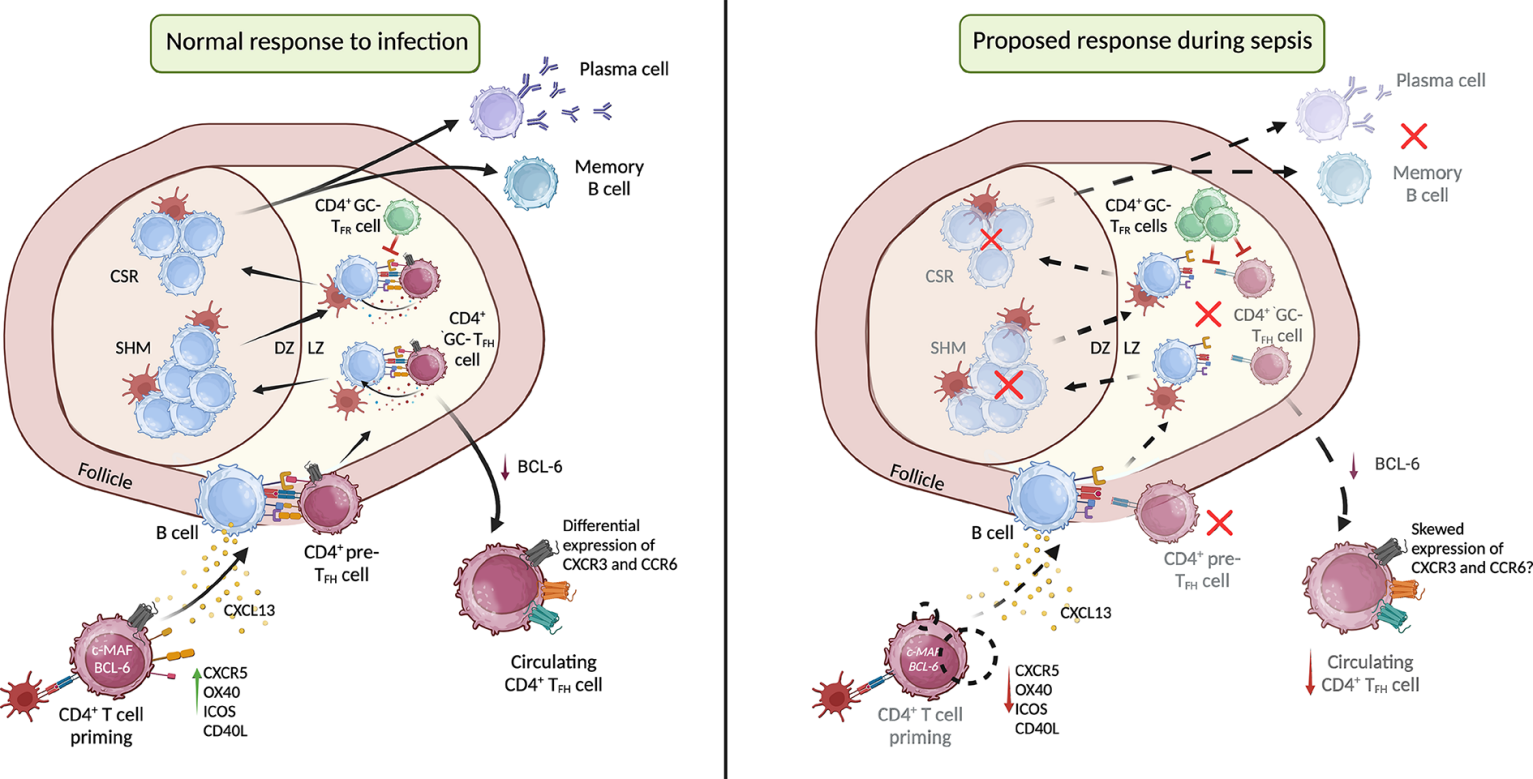
Suggested mechanisms of impaired CD4^+^ T_FH_ cell activity during sepsis During a normal response to infection (left panel), CD4^+^ T cells are initially primed by dendritic cells, inducing transcription of BCL-6 and subsequent expression of CXCR5 and other proteins important for migration to the B cell follicle, and generation of the germinal centre (GC). Within the GC, CD4^+^ T_FH_ cells provide signals (IL-21, IL-4, IL-10) to B cells for somatic hypermutation (SHM) and class-switch recombination (CSR), selecting those with highest affinity for antigen to differentiate into plasma cells or long-lived memory B cells. This process is regulated by CD4^+^ T_FR_ cells. GC-CD4^+^ T_FH_ cells may then down-regulate BCL-6 and enter the periphery as circulating memory cells, displaying different phenotypes through differential expression of CXCR3 and CCR6. During sepsis (right), multiple aspects of this process may be altered to result in inadequate B cell support. Suggested mechanisms include impaired transcription of c-MAF and BCL-6, resulting in reduced migration to the follicle to interact with cognate B cells. This could result in downstream effects of reduced numbers of GC-CD4^+^ T_FH_ cells with the correct protein expression profile needed to provide support. Alternatively, proliferation of CD4^+^ T_FR_ cells may result in enhanced suppression of GC-CD4^+^ T_FH_ cells. Both of these effects could result in a reduction in plasma cell differentiation and thus reduced antibody secretion. Alternatively, skewed expression of CXCR3 and CCR6 on circulating CD4^+^ T_FH_ cells could alter their cytokine signatures and subsequent ‘helper’ ability in the periphery. DZ: dark zone; LZ: light zone.

The underlying mechanisms driving changes in CD4^+^ T_FH_ cells that could explain defects in immunoglobulin secretion are poorly characterised. Conditions of persistent stimulation during severe bacterial and viral infections have been well-reported to drive ‘immunoparalysis’ in remaining T cells, describing an inability to mount or support an effective immune response [157]. In a study of the response to SARS-CoV-2 infection and vaccination, the neutralising antibody response robustly correlated with the frequency and phenotypic polarisation of circulating CD4^+^ T_FH_ cells [159]. Specific subsets of circulating CD4^+^ T_FH_ cells have been described, distinguished by their differential expression of the chemokine receptors CXCR3 and CCR6. Such subsets exhibit the behaviour of T_H_1, T_H_2 or T_H_17 cells, coined T_FH_1 (CXCR3^+^CCR6^−^), T_FH_2 (CXCR3^−^CCR6^−^), and T_FH_17 (CXCR3^−^CCR6^+^) cells respectively [160]. High titres of SARS-CoV-2 spike-specific or neutralising antibodies have consistently been associated with the frequency of T_FH_1 cells, with variability in reported relationships between antibody responses and T_FH_2 or T_FH_17 cells across studies [161–163]. The phenotype of circulating CD4^+^ T_FH_ cells has been reported for several other viral infections or vaccinations, with no clear consensus on an overarching subgroup best equipped for supporting antibody production. For example, T_FH_1 and T_FH_17 cells were found to predominate in non-responders to influenza virus vaccination, with a skewed IL-2/IL-21 axis incapable of supporting B cells [164]. In contrast, an increase in the frequency of T_FH_17 cells was demonstrated to correlate with enhanced antigen-specific antibody production following vaccination against Ebola virus [165]. Data in patients with human immunodeficiency virus (HIV) show a positive correlation between the frequency of T_FH_2 cells and the development of broadly neutralising antibodies, whilst T_FH_2 cells have been reported to impede an antiviral humoral response in chronic hepatitis B virus infection [166,167]. These varied findings potentially suggest a pathogen-specific aspect to the usefulness of different CD4^+^ T_FH_ cell subgroups in supporting B cells. Although many groups have reported skewing of CD4^+^ T_FH_ subsets in a virus-specific context, there are substantial gaps in the literature in the case of bacterial infections and sepsis. Based on the data, it seems clear that measurement of CD4^+^ T_FH_ cell frequencies in sepsis alone may be insufficient to explain a dampened ’helper’ response, and that phenotypic differences in CD4^+^ T_FH_ cells could alter their overall functional capacity. A separate study demonstrated impaired function of CD4^+^ T_FH_ cells in HIV-infected individuals, displaying downregulation of genes from immune- and GC-resident CD4^+^ T_FH_ cell-associated pathways including c-MAF and its upstream mediators [168]. These changes were associated with the resulting inefficient antigen-specific antibody response and death of memory B cells. Expression of c-MAF has been demonstrated as important in supporting BCL-6 expression in CD4^+^ T_FH_ cells following immunisation [169]. c-MAF and BCL-6 are crucial for upregulation of CD40L and ICOS expression on CD4^+^ T_FH_ cells as well as IL-21 signalling. Therefore, these transcriptional changes in HIV-infected individuals likely render CD4^+^ T_FH_ cells incapable of positioning themselves correctly within the GC to interact with and support their cognate B cells [169]. As HIV is a condition of chronic stimulation, it is plausible that sustained activation by high antigen load in sepsis could drive similar transcriptional changes in CD4^+^ T_FH_ cells, rendering them incapable of supporting B cell development. The inadequate help provided by CD4^+^ T_FH_ cells in HIV-infected individuals has sparked interest into the role of CD4^+^ T_FR_ cells in this context. In a study using an *ex vivo* model of tonsillar HIV infection and *in vivo* model of simian immunodeficiency virus infection in rhesus macaques, virus infection was associated with an expansion of suppressive CD4^+^ T_FR_ cells, expressing increased levels of co-inhibitory receptors CTLA-4 and lymphocyte-activation gene 3 (LAG-3), and increased production of anti-inflammatory cytokines IL-10 and TGF-β [170]. These cells were subsequently shown to impair CD4^+^ T_FH_ function through inhibition of cell proliferation and production of IL-4 and IL-21. The literature describing the role of CD4^+^ T_FR_ cells in sepsis is sparse, however, could provide important insight into functional changes to CD4^+^ T_FH_ cells if severe bacterial infections drive a similar expansion of CD4^+^ T_FR_ cells as seen in HIV infection. Further studies are required to determine if this is the case for sepsis, but also to expand our knowledge of CD4^+^ T_FH_ cell-mediated humoral immunity in the context of bacterial infections and sepsis (Figure 2).

## Alterations in other conventional and unconventional T cell types during sepsis

Sepsis-induced changes to T cells have been widely studied and implicated as important factors in determining the overall response and likelihood of survival. The sepsis-driven lymphopenia disproportionately targets the pool of antigen-inexperienced T cells in both mouse models and human studies [171,172]. This has been attributed to both a thymic defect affecting the output of newly generated T cells, and the acquisition of memory-like characteristics in otherwise naïve cells [173]. Such changes to the composition of the overall T cell repertoire contributes to increased susceptibility to secondary infections and may impair memory T cell generation [171,172]. In elderly patients, whose naive T cell pool is substantially reduced, destruction of this pool could cause long-term defects in mounting an effective immune response to new antigens [106,174]. Although naïve cells are particularly susceptible to sepsis-induced apoptosis and phenotypic changes, a numerical loss of existing memory CD4^+^ and CD8^+^ T cells has also been demonstrated [175,176]. Within the pool of memory CD4^+^ T cells, a preferential loss of ‘helper’ subpopulations including T_H_1, T_H_2 and T_H_17 cells shifts the balance towards a greater proportion of FOXP3^+^ T_REG_ cells [176–178]. T_REG_ cells represent a subset of CD4^+^ T cells implicated in negative immunomodulation, and the effects of their representative increase has been debated. Mouse models have demonstrated that the relative increase in T_REG_ cells is accompanied by an increased suppressive capacity. Indeed, T_REG_ cells were shown to suppress T cell proliferation to a greater degree in septic mice than those in sham-injured mice, with particular suppression of T_H_1-type cytokine production [179]. Additionally, T_REG_ cells induced apoptosis of monocytes and neutrophils in a CLP mouse model of sepsis through either Fas/FasL signalling or IL-10 secretion [180]. This enhanced suppression by T_REG_ cells has been correlated with worsened severity, however, other studies have correlated increased T_REG_ cell representation with an improved outcome and pathogen control [181,182]. Discrepancies may be due to timing of sample collection and infection course, with T_REG_ cells perhaps proving beneficial in patients experiencing overwhelming inflammation, whilst damaging in cases of immune exhaustion. T_REG_ cells have been suggested as a potential target for therapeutic intervention, however further analysis is necessary to determine approach [181,183].

The overall numerical reduction of CD4^+^ T cells is accompanied by functional defects, evidenced by increased rates of latent viral reactivation in septic patients [43,44,184,185]. A global, post-sepsis state of anergy has been proposed in CD4^+^ T cells, through evidence of little or no pro- or anti-inflammatory cytokine production evident following anti-CD3/CD28 stimulation in post-mortem spleen and lung samples [14]. Additionally, studies have shown a reduction in proliferative capacity and lineage-specific transcription factor expression, affecting the regulation of CD4^+^ T cell subset differentiation [172,186]. These observations are in line with increased co-inhibitory receptor expression such as PD-1 CTLA-4, LAG-3 and T cell immunoglobulin and mucin domain-containing protein 3 (TIM-3), altering how CD4^+^ T cells communicate with and modulate the responses of other immune cells [55,187]. In a normal immune response, T_H_1, T_H_2 and T_H_17 cells provide help to naïve CD8^+^ T cells to ensure a highly controlled and functionally specific response [36]. In addition, such signals promote clonal expansion upon re-encounter with antigen [188,189]. ‘Helpless’ T cells are instead destined for apoptosis. Decline of helper T cell populations during sepsis creates an environment in which CD8^+^ T cells could proceed to respond to antigen without CD4^+^ T cell help. This effect has been suggested to impair the early T cell effector response and contribute to a suppressive environment, through apoptosis of CD8^+^ T cells [188,189]. In addition, lack of CD4^+^ T cell help during primary infection results in memory CD8^+^ T cells which lack the capacity to respond during re-infection [36]. Memory CD8^+^ T cells from survivors are prone to exhaustion during chronic infection, with reduced capacity to secrete pro-inflammatory cytokines and increased expression of co-inhibitory receptors [171,190].

Research exploring sepsis-induced changes to T cells is largely focussed on conventional αβ T cells, with substantial gaps in the literature describing changes in unconventional T cell populations with antimicrobial functions, such as γδ T cells and mucosal-associated invariant T (MAIT) cells. As the first T cell population formed during embryonic development, γδ T cells constitute 0.5–5% of circulating CD3^+^ T cells in adult humans [191,192]. γδ T cells rapidly produce effector cytokines in response to bacterial infections and mediate protective immune responses against pathogenic microorganisms such as *Mycobacterium tuberculosis* (reviewed in [191]). Additionally, certain γδ T cells appear to possess potent antigen-presenting abilities during infections [193,194]. These unconventional T cells exist as two main populations in humans based on their encoded TCR δ-chain: Vδ1^+^ or Vδ2^+^ T cells. Vδ2^+^ T cells constitute the majority of peripheral blood γδ T cells whilst Vδ1^+^ T cells are less frequent in the blood and are more abundant in epithelial and mucosal tissues such as the skin, intestine and uterus [191,195–198]. In humans, the number of circulating γδ T cells decline in patients with sepsis compared to healthy controls, with an imbalance of pro- or anti-inflammatory functional changes depending on the subtype [199–201]. One study found an association between the degree of γδ T cell reduction and severity, whilst a separate study showed that impaired IFN-γ expression following *in vitro* antigen stimulation correlated with mortality [200,202]. Furthermore, the ability for γδ T cells to act as APCs is impaired during sepsis [203]. These sepsis-induced effects on γδ T cells appear to be specific to Vδ2^+^ T cells as it has been reported that peripheral Vδ1^+^ T cells increase in frequency during sepsis and correlate with increasing SOFA score and mortality [199]. Additionally, the expression of the co-inhibitory receptors CTLA-4 and TIM-3 were increased on these peripheral Vδ1^+^ T cells which are thought to possess an immunosuppressive function [199].

MAIT cells are ‘innate-like’ αβ T cell populations that make up 1-10% of all T cells in blood and mediate rapid, protective immune responses against bacterial species with intact riboflavin biosynthesis pathways, including *E. coli* and *S. aureus* [192,204–206]. MAIT cells use semi-invariant αβ TCRs to recognise ribityllumazine- and pyrimidine-based metabolite antigens from the riboflavin biosynthesis pathway, such as 5-OP-RU, that are presented by the non-classical MHC-like molecule, MR1 [207,208]. Such TCRs typically contain conserved usage of TCR α-chain variable gene 1-2 (TRAV1-2) paired with a biased pattern of TCR β-chain variable (TRBV) genes, such as TRBV20-1, TRBV6-4 or TRBV6-2/6-3 [204,209,210]. MAIT cell-deficient (*Mr1*^−/−^) mice demonstrate an enhanced susceptibility to bacterial infection [204] and increased mortality upon experimentally-induced sepsis [211]. Furthermore, this and other studies found reduced frequencies of MAIT cells in human patients with sepsis [211–214]. Whilst MAIT cells from these patients expressed more activation makers (e.g. CD69, CD38 and HLA-DR), they also exhibited higher levels of co-inhibitory receptors (e.g. LAG-3 and TIM-3) and were functionally deficient [211,212,214]. Indeed, in one study, such functional impairment of MAIT cells worsened over time during patient recovery from sepsis [212]. Furthermore, the phenotypic status of MAIT cells in sepsis patients may serve as a possible prognostic marker as the percentage of HLA-DR^+^ MAIT cells has been shown to be effective in predicting mortality and patient APACHE II scores [214]. Despite this knowledge, the impact of sepsis on MAIT cells and γδ T cells is poorly understood and also particularly understudied compared to more conventional αβ T cell populations. Data in mouse models of sepsis further illustrate the importance of MAIT cells and γδ T cells in modulating the host response to sepsis and their positive influence on survival [211,215]. Thus, further studies are required to expand our knowledge of sepsis-induced alterations in MAIT and γδ T cell immunity and to determine their utility as a prognostic biomarker or as a target for therapeutic intervention.

## Conclusions

Dysregulation of the adaptive immune system is a defining feature of sepsis, but the exact manifestation is widely variable between individuals. For this reason, developing novel therapeutics for sepsis has proved to be a challenge for over 30 years and, indeed, progress has been failing to meet the increasing demand as the burden of sepsis on hospitals worsens across the globe. A marked lymphopenia is a common feature across the literature; however, the phenotype of remaining cells is less well-defined. It is vital to develop a better understanding of the mechanisms underpinning the observed immune dysregulation to be able to suggest new targets for treatment or diagnostic biomarkers. Based on the diverse findings of several groups, it seems that considering sepsis as multiple separate conditions by grouping individuals displaying similar characteristics could show more promise for translating results to clinical practice. Patients frequently experience immunosuppression in some form during the course of sepsis, which can result in high susceptibility to secondary infections whilst hospitalised, and a decline in the long-term function of their immune system post-recovery. This may present as an impaired ability to produce high-affinity antibodies against pathogens, and as such may also have a negative impact on how individuals respond to vaccination post-sepsis. The relationship between CD4^+^ T_FH_ cells and B cells in sepsis remains to be thoroughly addressed, and also how the regulation of CD4^+^ T_FH_ cells by CD4^+^ T_FR_ cells is affected in this setting. Further work in this area could provide important insight into the decline in antibody production observed in many cases, and uncover new targets for treatment or modulation of the adaptive immune system long-term post-discharge from ICU.

## Data Availability

Data sharing is not applicable for this manuscript

## Open Access

Open access for this article was enabled by the participation of Cardiff University in an all-inclusive *Read & Publish* agreement with Portland Press and the Biochemical Society under a transformative agreement with JISC.

## Abbreviations

APACHE: Acute Physiology and Chronic Health Evaluation
APC: Antigen presenting cell
BCR: B cell receptor
B_REG_: B regulatory
CD40L: CD40 ligand
CLP: Caecum ligation puncture
CSR: Class Switch Recombination
CTLA-4: Cytotoxic T Lymphocyte-associated Antigen 4
*E. coli*: *Escherichia coli*
FO: Follicular
GC: Germinal Centre
HIV: Human Immunodeficiency Virus
HLA-DR: Human Leukocyte Antigen-DR
HSC: Haematopoeitic Stem Cell
ICOS: Inducible co-stimulator
ICU: Intensive Care Unit
IFN-γ: Interferon-γ
IL: Interleukin
IVIG: Intravenous immunoglobulin
LAG-3: Lymphocyte-activation gene 3
LPS: Lipopolysaccharide
MAIT: Mucosal-associated invariant T
MHC: Major Histocompatibility Complex
MZ: MarginalZ one
pAPCs: Professional antigen presenting cells
*S. aureus*: *Staphylococcus aureus*
SHM: Somatic hypermutation
SOFA: Sequential Organ Failure Assessment
TCR: T cell receptor
T_FH_: T follicular helper
T_FR_: T follicular regulatory
TGF-β: Transforming Growth Factor-β
T_H_: T helper
TIM-3: T cell immunoglobulin and mucin domain-containing protein 3
TNF-α: Tumour Necrosis Factor-α
TRBV: TCR β-chain variable
T_REG_: T regulatory
